# Voting-based integration algorithm improves causal network learning from interventional and observational data: An application to cell signaling network inference

**DOI:** 10.1371/journal.pone.0245776

**Published:** 2021-02-08

**Authors:** Meghamala Sinha, Prasad Tadepalli, Stephen A. Ramsey

**Affiliations:** 1 School of Electrical Engineering and Computer Science, Oregon State University, Corvallis, Oregon, United States of America; 2 Department of Biomedical Sciences, Oregon State University, Corvallis, Oregon, United States of America; Queen Mary University of London, UNITED KINGDOM

## Abstract

In order to increase statistical power for learning a causal network, data are often pooled from multiple observational and interventional experiments. However, if the direct effects of interventions are uncertain, multi-experiment data pooling can result in false causal discoveries. We present a new method, “Learn and Vote,” for inferring causal interactions from multi-experiment datasets. In our method, experiment-specific networks are learned from the data and then combined by weighted averaging to construct a consensus network. Through empirical studies on synthetic and real-world datasets, we found that for most of the larger-sized network datasets that we analyzed, our method is more accurate than state-of-the-art network inference approaches.

## Introduction

Causal modeling is an important analytical paradigm in action planning, predictive applications, research, and medical diagnosis [1, 2]. The main objective of causal modeling is to infer causal interactions in the form *V*_*i*_ → *V*_*j*_, where *V*_*i*_ and *V*_*j*_ represent observable entities and the direction of the arrow denotes that the state of *V*_*i*_ influences the state of *V*_*j*_. Causal models can be inferred from passive observational measurements (“*seeing*”) and also by measurements collected after performing external interventions (“*doing*”) on the states of the domain entities.

In many settings, observational measurements [3] are more straightforward to obtain than interventional measurements, and thus observational datasets are frequently used for causal inference. However, given only observational data, it is difficult to distinguish between compatible Markov equivalent models [4, 5]. For example, the three causal models *V*_*i*_ → *V*_*j*_ → *V*_*k*_, *V*_*i*_ ← *V*_*j*_ ← *V*_*k*_, and *V*_*i*_ ← *V*_*j*_ → *V*_*k*_, are Markov equivalent—each encodes the conditional independence statement *V*_*i*_ ╨ *V*_*k*_|*V*_*j*_. This ambiguity can in principle be resolved by incorporating measurements obtained from interventional experiments in which specific entities are targeted with perturbations. With the benefit of interventional measurements, Markov equivalent causal models can have different likelihoods, enabling selection of a maximum-likelihood model. These considerations have motivated the development of network learning approaches that are specifically designed to leverage mixed observational and interventional datasets [6].

Learning a causal network from a mixed observational-interventional dataset poses methodological challenges, particularly in integrating datasets from different experiments and accounting for interventions whose effects are uncertain [7]. Data collected from two different experiments might not be identically distributed and thus the two experiments may be incoherent from the standpoint of causal network model. For example, in molecular biology, “batch effects” can include differences in cellular growth conditions which can lead to experiment-specific effects on the joint distribution of the observables [8]. As a result, directly combining data from different experiments can lead to errors in network learning. Interventions, too pose a challenge due to the fact that in real-world settings many interventions are (i) imperfect, meaning interventions are unreliable and have soft-targets (A “soft” target intervention, or “mechanism change,” is an intervention that changes a target node’s distribution’s parameters, but does not render that it’s independent of its parent nodes [7]), and (ii) uncertain, meaning that the “off-target” nodes are unknown. Classical causal learning algorithms are based on the assumption that interventions are *perfect* [1]; applying such algorithms to a dataset derived from imperfect interventions would likely yield spurious interactions. Eberhardt [9] classifies such errors into two types: a) *independence to dependence* errors, where two variables *V*_*i*_ and *V*_*j*_ that are independent are detected as dependent when data from the observational and interventional experiments are pooled (i.e., false positive detection of a causal interaction) and b) *dependence to independence* errors, where two variables *V*_*i*_ and *V*_*j*_, that are dependent in an observational study are independent when the data from the observational study are pooled with data from an interventional study (i.e., a false negative for the interaction). Consensus has yet to emerge on the question of how—given two or more datasets generated from different interventions—the datasets should be combined to minimize such errors in the learned network model. Another similar problem can also arise where datasets from different experiments have overlapping but non-identical set of variables. This problem has been described in the context of a neuroimaging dataset [10], where different regions of interest (ROIs) in the brain can be imaged for different individuals; not taking into account these discrepancies can cause erroneous results (the authors of [10] proposed the IMaGES algorithm to address this issue). More generally, the problem of overlapping variables and how to cluster them was considered in [11]. However, in this work we have only considered experiments having identical non-overlapping variables.

In this paper, we report the results of a multi-dataset analysis of the performance of our proposed method, “Learn and Vote” [12], for inferring causal networks from multi-experiment datasets. “Learn and Vote” can be used to analyze datasets from mixed observational and interventional studies and it is compatible with uncertain interventions. As it is fundamentally a data integration method, “Learn and Vote” is compatible with a variety of underlying network inference algorithms; our reference implementation combines “Learn and Vote” data integration with the Tabu search algorithm [13] and the Bayesian Dirichlet uniform (BDeu) [6, 14, 15] network score, as described below. Use of Learn and Vote produces a weighted causal graph, where each edge has an associated weight (in terms of probabilistic measure) for its strength and direction. To characterize the performance of “Learn and Vote”, we empirically analyzed the network learning accuracies of “Learn and Vote” and six previously published causal network learning methods (including methods that are designed for learning from heterogeneous datasets) applied to six different network datasets. Of the six network datasets, the largest real-world dataset is a cell biology-based, mixed dataset (the Sachs et al. dataset [16]) with a known ground-truth network structure. On larger networks, we report superior (or in worst case, comparable) performance of “Learn and Vote” to the six previously published network inference methods.

## Motivation and background

### Spurious dependencies and independencies

**Fig 1:**
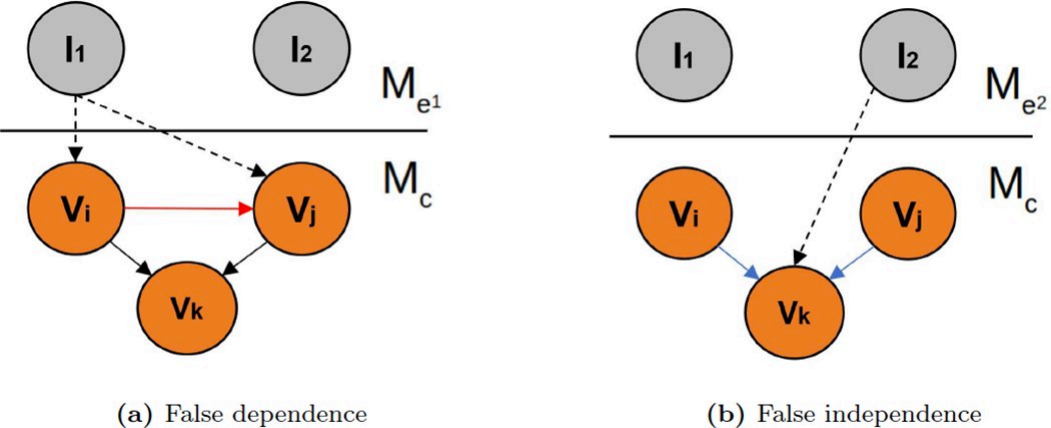
Cross-experiment data pooling leads to network inference errors. Illustration of a simple hypothetical causal model *M*_*c*_ with three observable entities (*V*_*i*_, *V*_*j*_, and *V*_*k*_. Two different interventional experiments are depicted: experiment $M_{e^{1}}$ involves intervention *I*_1_, and experiment $M_{e^{2}}$ involves intervention *I*_2_. Pooling measurements from the two experiments can cause two types of network inference errors: false positive edge (shown in (a) as a red arrow between *V*_*i*_ and *V*_*j*_), and false negative edges (shown in (b) as blue arrows between *V*_*i*_ and *V*_*k*_ and between *V*_*j*_ and *V*_*k*_).

In this section, we introduce notation and describe how spurious dependencies or independencies can occur when we perform perturbations affecting two or more variables in a causal model. Mathematically, a causal model *M*_*c*_ is described by a directed acyclic graph (DAG) containing a pair (*V*, *E*), where *V* is a set observable nodes (corresponding to random variables), *E* represents set of directed edges between two nodes, Pa(*V*_*i*_) represents the set of parent nodes of variable *V*_*i*_, and *P*(*V*) represents the joint probability distribution. In the context of network learning from interventional data, it is helpful to picture an intervention (say, *I*_1_) as a separate type of node (denoted by a dashed circle in Fig 1) that can be connected to its targets (say, *V*_*i*_ and *V*_*j*_) by causal edges of a separate type (dashed arrow in Fig 1). Applying classical network inference algorithms to measurements pooled from multiple interventional experiments can lead to two different types of learning errors, as we explain below.

1. **False causal dependence**: In the experiment depicted in Fig 1a, *V*_*i*_ and *V*_*j*_, which are not causally related in *M*_*c*_ (*V*_*i*_ ↛ *V*_*j*_), are affected by intervention *I*_1_. Due to the intervention’s confounding effect, we have 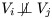 in the combined model $M_{T_{1}}=M_{c}+M_{e^{1}}$ (we denote the joint distribution in the combined model by $P_{1}(\text{V}\subset M_{T_{1}})$). Thus, spurious correlations between independent variables may occur if we pool data from such different distributions.
2. **False causal independence**: In the experiment depicted in Fig 1b, the intervention *I*_2_ on *V*_*k*_ removes all the incident arrows for *V*_*k*_ and cuts off the causal influences of *V*_*i*_ and *V*_*j*_ on *V*_*k*_, causing *V*_*i*_ ⫫ Pa(*V*_*i*_). Pooling data from such models can cause the causal dependencies *V*_*i*_ → *V*_*k*_ and *V*_*j*_ → *V*_*k*_ in *M*_*c*_ to be missed (i.e., a “false negative” in the inferred network).

### Review of prior literature

A causal network works like a Bayesian Network with similar applicability (e.g., intelligent systems [17, 18], recommendations, cognition [19, 20], medical diagnosis [21] etc) except that the relationships should be causal. Classical causal learning methods fall into two classes: *constraint-based* methods (e.g., PC [2], FCI [22]), in which the entire dataset is analyzed using conditional independence tests; and *score based* methods (e.g., GES, GIES [23]), in which a score is computed from the dataset for each candidate network model. Both classes of methods were designed to analyze a single observational dataset, with the attendant limitations (in the context of multi-experiment datasets) that we described above. Several multi-dataset network inference approaches have been proposed that circumvent the above-described problems associated with cross-experiment measurement pooling. Cooper and Yoo [6] proposed a score-based algorithm that combines data from multiple experiments, each having perfect interventions with known targets. The approach was later refined by Eaton and Murphy [7] for uncertain and soft interventions [24]. The method of Claassen and Heskes [25] is based on imposing the causal invariance property across environment changes. Sachs et al. [16] analyzed a molecular biology dataset (which has since become a benchmark dataset for molecular network inference, a primary application focus of our work) using a variant of the Cooper-Yoo method. Chen et al. [26] proposed a subgraph-constrained approach, called Trigger, to learn a yeast gene regulatory network model from transcriptome and genotype data. In the Joint Causal Inference (JCI) [27] method, additional experimental context variables are introduced before data pooling. Notably, the aforementioned methods make some assumptions about the network model, for example: whether the interventions are “perfect” or whether any “context” variable can be defined to differentiate the data. The “Learn and Vote” method (see Methods and datasets) is designed for the situation where one cannot make any such assumptions about the underlying model in each experiment.

#### Network combination methods

Another class of multi-dataset network inference approaches, which we call “network combination” methods, involve learning causal interaction statistics from each experiment followed by integration of the statistics to obtain a single consensus network. For example, in the ION [11] method, locally learned causal networks having overlapping variables are integrated. The constraint-based COmbINE [28] method is based on the estimation of variable-variable dependencies and independencies across separate experiments. The MCI [29] algorithm is another example of a constraint-based method that utilizes the ‘local’ aspect of V-structures (as defined in [30] a V-structure is a triple of variables (X,Y,Z) such that there are converging arrows from X and Y on Z and there is no link between X and Y) [31]. However, none of these methods produce experiment-specific weighted graphs (which an edge’s weight representing degree of confidence), instead enumerating experiment-specific partial ancestral graphs that are consistent with the data. In real-world datasets, due to a variety of factors (finite sampling, experiment-specific biases and confounding effects, measurement error, missing data, and uncertain/imperfect interventions), the confidence with which a given causal interaction *V*_*i*_ → *V*_*j*_ can be predicted within a given experiment will in many cases vary significantly from experiment to experiment (and in the case of incomplete measurements, may not be quantifiable at all in a given experiment). Thus, a network combination method compatible with experiment-specific edge weights would seem to offer a distinct advantage in the context of multi-experiment network inference. Furthermore, all of these methods assume that a single underlying causal model accounts for all observed causal dependencies. In real-world settings where experimental conditions change across experiments, this assumption seems unlikely to hold, motivating the need for network inference methods that can (1) score candidate interactions within individual experiment-specific datasets and (2) combine weighted edges from experiment-specific datasets into a consensus network.

### Biological signaling networks

**Fig 2:**
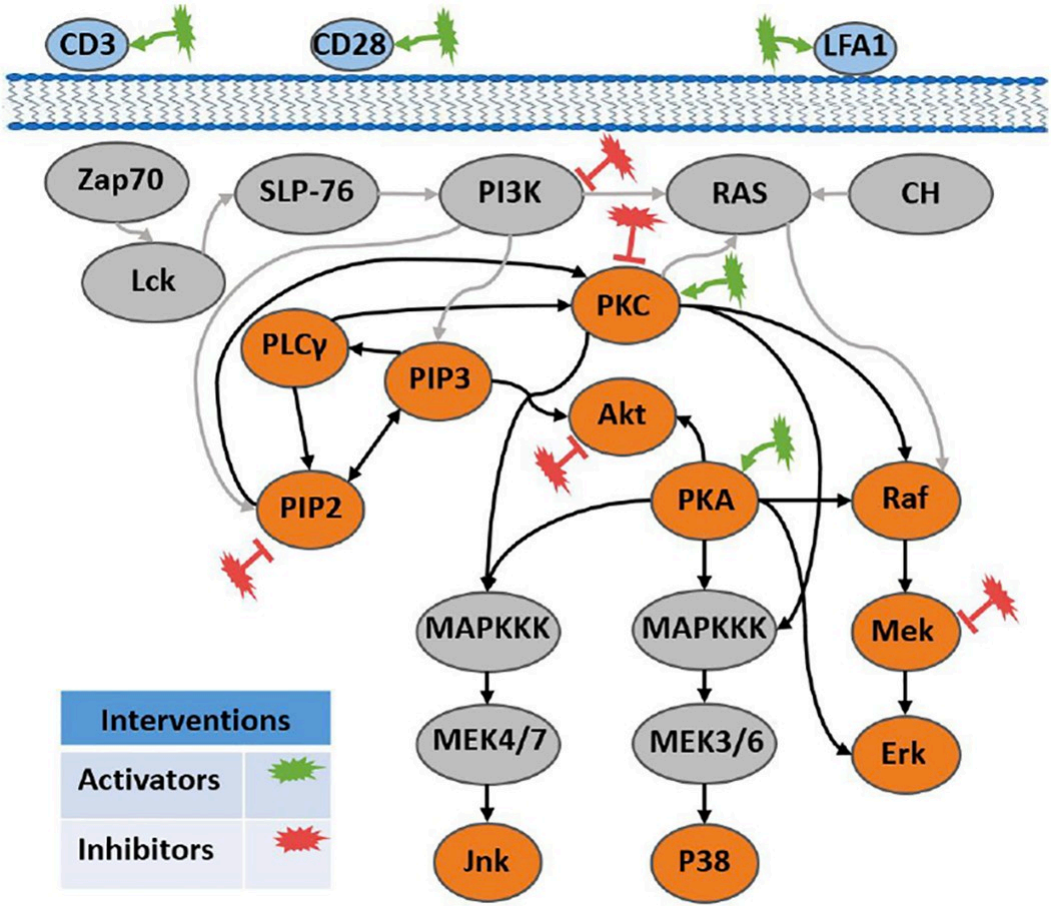
Biological network for the Sachs et al. study, showing interactions (arcs) and interventions (starred ellipses). The pathways represented by bold black lines are the Ground Truth known causal interactions, established through literature study.

A cell signaling network is a type of causal network in which the state of a protein or other biomolecule influences the state of another protein or biomolecule downstream of it (denoted by a directed arc). Such networks are amenable to interventional experiments using molecular agents that target (i.e., activate or inhibit) specific molecules. Sachs et al. [16] used a Bayesian network approach to infer causal interactions among eleven signaling molecules in human CD4+ T-cells. In a series of nine experiments—two observational and seven with specific molecular interventions—they used flow cytometry to measure the levels of activation of eleven phosphorylated proteins and phospholipids in individual cells (Fig 2). They inferred a network containing 17 true positive interactions among which 15 were well-established through literature survey of biology publications and two that were supported by at least one study; their inferred network missed three arcs (false negatives) and it had no false positive arcs.

Insofar as it involves issues of how to analyze multi-experiment datasets that were acquired under uncertain interventions, data-driven biological network reconstruction is an archetype causal inference application that clearly illustrates the problems inherent to data pooling. We found the Sachs et al. cell signaling dataset to be an ideal test-bed for developing a multi-experiment analysis method for causal inference, due to its nontrivial network size, expert-curated ground-truth network, and relatively large number of interventions.

### Uncertain interventions

In the network analysis method used in the Sachs et al. study and in our re-analysis of the Sachs et al. data, interventions were assumed to be “perfect”, i.e., each of the interventional agents was assumed to target exactly one of the signaling molecules. Such a perfect intervention assumption is likely not consistent with typical interventions in biological systems, due to potential off-target effects of pharmaceutical agents. Moreover, in a biological system, the effects of certain types of interventions (for example, a gene knockout) may not be describable by forcing of a target node’s state to a specific value in the observational network. In the Sachs et al. experiments, even though the authors have assumed that the interventions are perfect, they actually have off-target effects, as demonstrated by Eaton & Murphy (2007) [7]. Eaton & Murphy modeled chemical interventions as context variables in the network (assuming they had some known background knowledge about the underlying network) to learn the intervention’s effects and found them to have multiple children. To summarize, in the context of current learning algorithms, there are three primary issues with pooling experimental data that were acquired with imperfect interventions:

1. Current algorithms might make mistakes since the arcs pointing towards the unknown targets are not removed or handled properly.
2. Although pooling data adds more confidence into learning the true causal arcs, it can also introduce spurious arcs with incorrect direction (see Fig 4).
3. Each intervention might alter a mechanism or influence the local distribution in an unknown way [24].

## Methods and datasets

**Fig 3:**
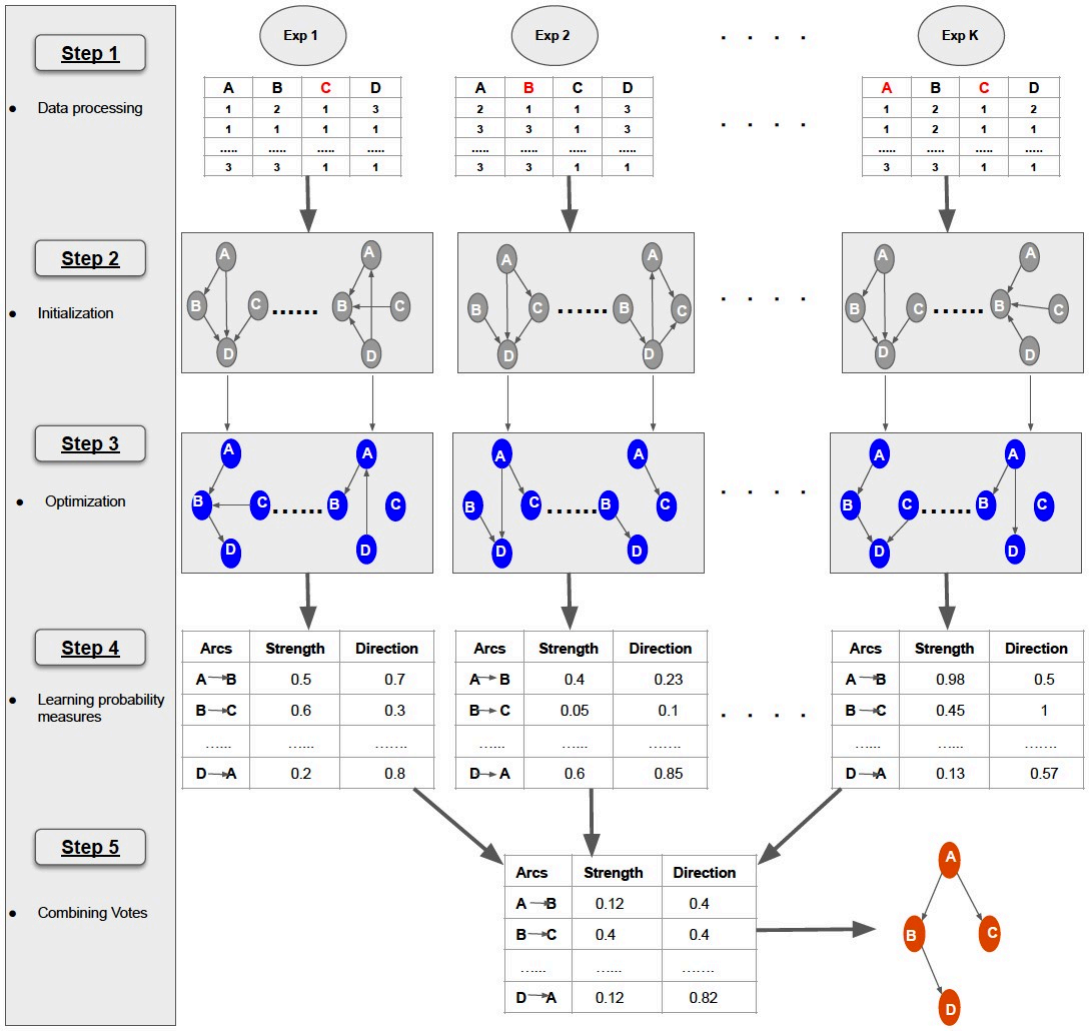
Workflow of “Learn and Vote”. **Step 1**—Collecting data from *k* experiments (combination of observational and interventional studies). For interventional studies, the known targets (marked in Red) are incorporated as external perturbation during the search process. **Step 2**—Creating 100 random DAGs using the observed nodes, as a starting point. **Step 3**—Optimizing each of the 100 DAGs with data using Tabu search. **Step 4**—Calculating probability (in terms of strength and direction) of occurrence for every possible arc from the 100 optimized DAGs and storing them in tables. **Step 5**—Combining votes from all the tables by weighted averaging and constructing the final causal network, with arc strengths above a threshold (in this case 50%).

To avoid the problems arising from pooling data from different experiments in causal network learning, we propose the “Learn and Vote” method (shown in Fig 3 and Algorithm 1). The method’s key ideas are enumerated as follows:

1. Suppose there are *k* experiments (which can include both observational and interventional experiments) that produced *k* datasets.
2. For interventional experiments in which the interventions’ targets are known, we use an experiment-specific modified scoring function in which the arcs that the targets’ incident arcs are deleted. However, we assume that each intervention might also have additional “off-target” effects which are unknown.
3. From each experiment, learn a weighted network in which each (possible) edge has a probability score, via the following steps:
4. For each experimental dataset, we create 100 random connected DAGs.
5. Run Tabu 100 times, each time using one of the 100 DAGs as starting graph, to learn 100 optimized DAGs.
6. Using the 100 optimized DAGs, compute the probability of the strength and direction of each arc as its empirical frequency of occurrence among the DAGs. For example, if an edge *X* → *Y* appears in 90 out of 100 optimized DAGs, it is assigned probability 0.90. Store each experiment’s arc weights in a list.
7. Repeat step 4-6 for all the *k* experiments.
8. From the *k* arc-weight lists, average arc strengths and directions over all the *k* experiments in which the given arc is valid (i.e., for which the arc’s target node is not intervened).
9. Finally, from the averaged arc strengths, we apply a threshold (0.5) over the probabilities and build the final causal DAG.

We provide psuedocode for our method in Algorithm 1. The algorithm’s inputs are, for each experiment, the observed variables (*V*) in the experiments (we denote the number of variables by *v* and the number of experiments by *k*) and the identities of the known target nodes (stored as a list *INT*) for any interventions.

**Algorithm 1** The Learn and Vote algorithm

**Input**: Datasets *D*_1_,*D*_2_…*D*_*k*_ each collected from experiments 1,…,*k*

**Output**: The final constructed causal network DAG *G*^*f*^ = (*E*, *V*)

1: **procedure** Learn and Vote

2:  **for** *j* from 1 to *k* **do**

3:   *V* = nodes or columns in dataset *D*_*j*_

4:   INT = Intervened nodes in dataset *D*_*j*_ for experiment *j*

5:   randomNet = createRandomNet(*V*, 100)

6:   **for** *m* from 1 to 100 **do**

7:    Net[*m*] = Tabu(randomNet[*m*], INT)

8:   arcProbability[*j*] = arcStrength(Net)

9:  averageArcs = averageNetwork(arcProbability)

10:  *G*^*f*^ = learnDAG(averageArcs,Threshold)

### Scoring function

We incorporate the effect of intervention in the score component associated with each node by modifying the standard Bayesian Dirichlet equivalent uniform score (BDeu) [6, 14, 15]. Given measurements *D*_*j*_ of variables *V* in experiment *j*, let *G*^*j*^ represent a DAG learned from it (with conditional distributions $P(V_{i}|\text{Pa}{\left(V_{i}\right)}^{G^{j}})$, where $\text{Pa}{\left(V_{i}\right)}^{G^{j}}$ represents the set of parent nodes of *V*_*i*_ in DAG *G*^*j*^). In a perfect interventional experiment, for the set INT(*m*) of intervened nodes in sample *m*, we fix the values of *V*_*i*_[*m*] ∈ INT(*m*), meaning that we exclude *P*(*V*_*i*_[*m*] | Pa(*V*_*i*_)[*m*]) from the scoring function for *V*_*i*_ ∈ INT(*m*). All the other unaffected variables are sampled from their original distributions. The distribution of *D*_*j*_ is per experiment and not a pooled dataset of all experiments as in the Sachs et al. method. We define an experiment-specific network score *S*(*G*^*j*^: *D*_*j*_) as sum (over all variables *V*_*i*_) of per-variable local scores *S*_local_(*V*_*i*_, *U*: *D*_*j*_) of variables *V*_*i*_. The left part of the equation is the prior probability assigned to the choice of set *U* as parents of *V*_*i*_, and the right part is the probability of the data integrated over all possible parameterizations *θ* of the distribution,

$$\begin{array}{c} S_{\text{local}}\left(V_{i},U:D_{j}\right)=\text{log}\;P\left(Pa_{i}=U\right)+\text{log}\;\int \prod_{m,V_{i}\notin \text{INT}\left(m\right)}P\left(V_{i}\left[m\right]|U\left[m\right],\theta \right)dP\left(\theta \right). \end{array}$$

### Structure learning

Because our method a uses local stochastic search algorithm (Tabu), we create an ensemble of *n* random starting DAGs (stored as randomNet, see Algorithm 1) using the procedure createRandomNet. Empirically, we have found that *n* = 100 is adequate for the network multivariate datasets that we analyzed in this work to demonstrate empirical performance of our method (see Results). From each DAG in randomNet, we then search for an optimal network model using the Tabu search algorithm [13] and store the *n* networks in a list Net. We chose Tabu because it is quite robust, simple (few parameters), efficient, and has a history-dependent (“memory”) to avoid cycling. We found that it performed well, although a systematic comparison to other optimization methods was not the goal of this study. The list INT which incorporates interventions on the known targets is passed as an argument to the search algorithm. This prevents the arcs from being incident on the targets. Next, we use the procedure arcStrength to measure the probabilistic arc strength along with its direction (for each arc) as its empirical frequency using the list of networks in Net. Finally, we average the arc strengths for every possible directed arc over the networks in which corresponding target node was not intervened. We store these measurements as a list called arcProbability.

### Combining results from experiments

We use the derived arc information stored in arcProbability (see Algorithm 1) and compute the average of arc strengths and strength of their directions over the number of experiments in which the given arc is valid (using procedure averageNetwork). Next, to construct the final causal DAG (using procedure learnDAG) we compute the averaged arc strengths as averageArcs and use a predefined Threshold over them. We found that our method performs best with 50% threshold. We implemented “Learn and Vote” in the R programming language, making use of the bnLearn package [32]. Software implementations of the sub-procedures used in the algorithm are provided in the Learn and Vote source code repository github.com/meghasin/Learn-Vote.

#### Datasets that we used for empirical performance analysis

From six published networks, we obtained nine datasets (with associated ground-truth networks) that we analyzed in this work. For each network we used both observational and interventional datasets. For synthetic networks, as observations, we drew random samples (1,000 samples per experiment). As interventions, we set some target nodes to fixed values. We model uncertain interventions by setting one or more children of the (known) target node to different values (simulating a “fat-hand” type of intervention [7] whose off-target effects are unknown). Interventions, for the synthetic dataset, were chosen at random. But we avoided intervening the leaf nodes (we can make this choice since we know the structure of each network), because ideally leaf nodes will not cascade any causal flow of information. However, from real world data its difficult to identify the most informative points of target. The targets and their uncertain effects, for each network, are selected as shown in our source code in the Data Availability section. Next, from each of these new mutilated networks [33], we sample a fixed (equal) number of observations from each experiment. A brief description of the six networks from which we collected and analyzed data are as follows:

- **Lizards**: This is a real-world dataset with three variables illustrating the perching behaviour of two different species of lizards from South Bimini island [34]. For our study we created two mutilated networks (with fat-hands) and sampled two interventional datasets, one from each. We also use one observational dataset from the lizards network. All these datasets have equal sample size.
- **Asia**: This is a synthetic network of eight variables [35] representing occurrence of lung diseases in human and their connection with whether they visited Asia or not. We created two mutilated networks Asia_mut1 and and Asia_mut2 by intervening at different nodes (to see how intervening at different targets affects performance). The experiment from Asia_mut1 has one observation and one interventional dataset, and the experiment from Asia_mut2 has one observational and two interventional datasets.
- **Alarm**: This is a synthetic network containing thirty seven variables characterizing the mechanism of an alarm messaging system used to monitor patients [36]. For our empirical study, we created two mutilated networks Alarm_mut1 and Alarm_mut2 as described above. The experiment Alarm_mut1 has three observational and six interventional datasets, and the experiment Alarm_mut2 has five observational and ten interventional datasets.
- **Insurance**: This is a synthetic network with twenty seven variables to evaluate car insurance risks [37]. We created two mutilated networks Insurance_mut1 and Insurance_mut2. For the experiment Insurance_mut1 we obtained one observational and five interventional datasets; and for Insurance_mut2, we obtained three observational and eight interventional datasets.
- **gmInt**: This is a synthetic dataset which is a matrix containing mix of observational and interventional data from eight Gaussian variables. This dataset is available in the pcalg-R package.
- **Sachs et al**.: a cell signaling network and associated mixed observational-interventional dataset published by Sachs et al. [16], described above).

The datasets described above are publicly available online at github.com/meghasin/Learn-Vote/tree/master/data

#### Causal network learning methods that we compared to “Learn and Vote”

Using the aforementioned networks and datasets, we compared the accuracy of “Learn and Vote” for network inference to the following six algorithms (implemented in R):

- **PC**: For this method we only used the observational datasets to evaluate DAG-equivalent structures [2]. In this study we used the Fisher’s *z*-transformation conditional independence test, with *α* value 0.01. We used the pcalg-R package for the implementation of this method.
- **GDS**: GDS (Greedy DAG Search) is a type of greedy search methods [23] which are used to estimate Markov equivalence class of DAG from data (observational and interventional). This method works by maximizing a scoring function (*L*_0_-penalized Gaussian maximum likelihood estimator) in three phases, i.e., addition, removal and reversal of an arc in the network, till the score is improving. We used the pcalg-R package for the implementation of this method.
- **GIES**: GIES (Greedy Interventional Equivalence Search) is another type of greedy algorithm [23] which extends the greedy equivalence search (GES) algorithm [38] so that it is possible to include interventional data into observational data. We used the pcalg-R package for the implementation of this method.
- **Globally optimal Bayesian network (simy)**: Simy is a function of a score-based dynamic programming approach [39]. We used the pcalg-R package for the implementation of this method. implemented to find the optimum of any decomposable scoring criterion (examples BDe, BIC, AIC). This function evaluates the best Bayesian network structure given a mix of interventional and observational data. However, this method is only feasible for networks containing up to about twenty variables.
- **Invariant Causal Prediction (ICP)**: This is a method proposed by Peters et al., [40]. The idea is to exploit the invariance property of a causal (vs. non-causal) relationship under different experimental settings and calculate the confidence intervals for those causal effects. We used the R package InvariantCausalPrediction for our study.
- **Sachs et al. method** This is the Bayesian network approach used by Sachs et al. as we described in Methods and Datasets above. We used the bnlearn-R package in the implementation of this method.

For each of these methods except PC, the method implementations that we used were adapted for heterogeneous datasets (see citations above).

### Performance measurement

For the purpose of quantifying the accuracies of the nine networks learned by each of the seven network algorithms, we considered the occurrence of an arc in the ground-truth network as a “positive” and the absence of an arc as a “negative”. For each of the final inferred causal network and each of the algorithm, from the confusion matrix we computed precision, recall, and the F1 harmonic mean of precision and recall (we did not compute accuracy due to the inherent class imbalance of sparse networks), as shown in Table 1.

**Table 1:** Multi-dataset performance of “Learn & Vote” versus six other methods. Each row corresponds to a specific dataset derived from a specific underlying ground-truth network (as described in detail in Methods and Datasets). Each row is split into three structure learning performance metrics (precision, recall, and “F1” score, harmonic mean of precision and recall). For each sub-row, the highest performance measurement is boldfaced. Each column corresponds to a specific method for causal network inference (as described in detail in Methods and Datasets), with the performance measures of our method (“Learn and Vote”) in the rightmost column. The symbol “n/a” denotes that no performance results were available for that method on that dataset. Here, the method “simy” is only feasible for networks containing up to 20 nodes, so it failed to produce results on the larger networks. The network **size** denotes the number of nodes in the indicated network. The network **type** is as follows: RW, real-world; S, synthetic.

Dataset	size	type	Metric	PC	GDS	GIES	ICP	simy	Sachs et al.	Learn & Vote
**Lizards**	3	RW	Precision	**1**	**1**	**1**	0	**1**	**1**	**1**
			Recall	**1**	**1**	**1**	0	**1**	0.5	0.5
			F1 score	**1**	**1**	**1**	0	**1**	0.667	0.667
**Asia_mut1**	8	S	Precision	**1**	0.625	0.625	**1**	0.316	0.77	**1**
			Recall	0.75	0.625	0.625	0.5	0.75	**0.875**	0.75
			F1 score	**0.857**	0.625	0.625	0.666	0.444	0.824	**0.857**
**Asia_mut2**	8	S	Precision	**1**	0.857	0.857	**1**	0.304	0.666	**1**
			Recall	0.75	0.75	0.75	0.5	**0.875**	0.75	0.75
			F1 score	**0.857**	0.8	0.8	0.666	0.493	0.706	**0.857**
**gmInt**	8	S	Precision	0.75	0.889	0.889	**1**	0.889	0.857	**1**
			Recall	0.75	**1**	**1**	0.375	**1**	0.75	0.75
			F1 score	0.75	**0.94**	**0.94**	0.545	**0.94**	0.8	0.857
**Cell signaling**	11	RW	Precision	0.571	0.419	0.377	**1**	0.422	0.68	0.89
			Recall	0.4	**0.9**	0.85	0.45	0.95	0.85	0.89
			F1 score	0.47	0.572	0.522	0.62	0.584	0.756	**0.89**
**Insurance_mut1**	27	S	Precision	0.714	0.36	0.362	0.7	n/a	**0.857**	0.8
			Recall	0.288	0.346	0.327	0.25	n/a	**0.577**	0.538
			F1 score	0.411	0.352	0.343	0.368	n/a	**0.689**	0.643
**Insurance_mut2**	27	S	Precision	**0.714**	0.355	0.366	0.64	n/a	0.676	0.686
			Recall	0.288	0.423	0.423	0.21	n/a	0.442	**0.461**
			F1 score	0.411	0.386	0.392	0.316	n/a	0.535	**0.552**
**Alarm_mut1**	37	S	Precision	0.666	0.25	0.26	**0.7**	n/a	0.625	0.564
			Recall	0.434	0.217	0.26	0.26	n/a	**0.446**	0.4
			F1 score	**0.526**	0.232	0.26	0.38	n/a	0.52	0.468
**Alarm_mut2**	37	S	Precision	0.666	0.411	0.513	0.6	n/a	0.725	**0.769**
			Recall	0.434	0.456	0.434	0.21	n/a	0.63	**0.642**
			F1 score	0.526	0.432	0.47	0.311	n/a	0.675	**0.7**

## Results

### Effect of interventions on network inference

**Fig 4:**
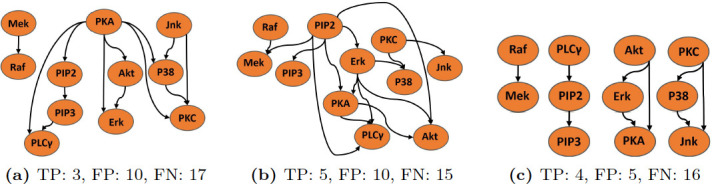
Networks inferred by (a) pooling data from two observational experiments; (b) pooling data from an observational (anti-CD3/CD28) and an interventional experiment (AKT inhibitor); and (c) our method “Learn and Vote” analysed on the same experiments as in the middle panel (b). The metrics used for structure learning evaluation are True Positive (TP), False Positive (FP) and False Negative (FN). False positives are reduced by avoiding pooling.

Based on prior studies suggesting that incorporating data from interventional experiments improves network inference (see Introduction), we re-analyzed a small subset of the Sachs et al. [16] biological cell signaling dataset (for which a ground truth network was published [16]) using their published inference approach, two times. First we used only two observational experiments (Fig 4a) having 600 samples each and second we used one observational and one interventional experiment (Fig 4b) having 600 samples in each. We found that sensitivity for detecting cell signaling interactions increases when data from observational and interventional experiments are co-analyzed (Fig 4b), versus when only data from observational experiments are used (Fig 4a). These results illustrate the benefit of using data from interventional experiments for causal network reconstruction.

### Effect of pooling on network inference

Based on prior studies suggesting that pooling data from multiple experiments can lead to errors in network learning (see Introduction), we analyzed the same cell signaling dataset as in Fig 4b, using the “Learn and Vote” method, in which data are not pooled. Compared to the the Sachs et al. inference method which was based on data pooling (Fig 4b), use of “Learn and Vote” significantly reduced false positives, while increasing the overall robustness of the network learning (Fig 4c). The overall poor performance in Fig 4a and 4b is because by using data from only two experiments in each, the total data size was in each case only 1,200 as compared to 5,400 in the original Sachs et al. study.

### Systematic comparative studies

To study the performance characteristics of “Learn and Vote” for a broader class of network inference applications, we carried out a systematic, empirical comparison of our method’s performance with six previously published causal network learning methods using nine datasets (from six underlying networks of small to medium size, as described above in Methods and Datasets), spanning a variety of application domains.

#### Networks learned by the seven methods on the cell signaling dataset

**Fig 5:**
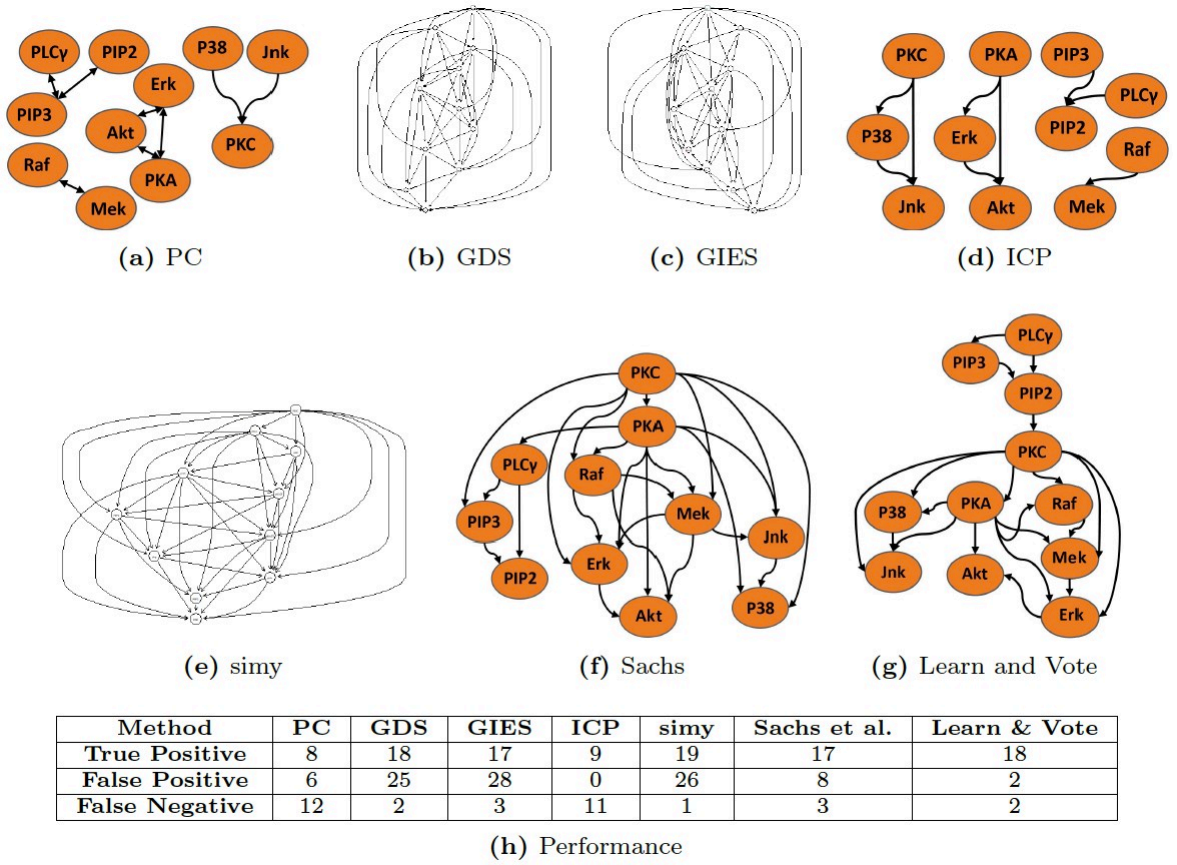
Consensus networks inferred from various algorithms (a-g) on the Sachs et al. cell signaling dataset. A bidirectional arrow between two nodes denotes that an interaction is predicted between the two nodes, but the direction of causality is ambiguous. In the table (h), each row corresponds to a component of the confusion matrix (true positives, false positives, and false negatives), and each column corresponds to a causal network inference method.

On the Sachs et al. dataset, the consensus networks that each algorithm learned are shown in Fig 5a–5g; the networks varied significantly in terms of density, with GDS, GIES, and simy giving large numbers of edges, and PC and ICP giving relatively sparse networks (with the PC network having many ambiguous arc directions). For each of the methods, we tabulated the numbers of correct and incorrect (or missing) arcs in the consensus networks learned (Fig 5h). The greedy algorithms (Fig 5b and 5c) and simy (Fig 5e) were able to infer most of the true positive arcs but there was a large number of false positives detected. The consensus “Learn and Vote” network (Fig 5g) improved over the consensus network obtained using the Sachs et al. inference method (Fig 5f), by eliminating six false positive edges and gaining a true positive edge (*PIP*2 → *PKC*) (Fig 5h, rightmost two columns). We further note that two of the putatively false interactions that were detected by “Learn and Vote”, (*P*38 → *pjnk*) and (*PKC* → *Erk*), on further study through PCViz (www.pathwaycommons.org/pcviz) and PubMed (www.ncbi.nlm.nih.gov/pubmed) are found in literature and hence likely interactions. Moreover, our method had the lowest number of false positives among all seven methods and was tied for second-highest in terms of the number of true positives (Fig 5h).

### Quantifying performance of seven network learning algorithms

In Table 1, we summarize the performance, in terms of network learning precision, recall, and F1 score of the seven network inference methods applied to nine datasets (with associated ground-truth networks) that were described in Methods and Datasets. In terms of F1 accuracy, while the PC algorithm (which used *observational* measurements) has strong performance on smaller networks, “Learn and Vote” has superior performance for learning the structure of larger networks. More broadly, in terms of precision, “Learn and Vote” outperformed the other six algorithms in five out of nine dataset. The ICP method have the second best performance. The positive predictive rate of “Learn and Vote” is higher for small and medium networks (i.e., networks with fewer than 20 nodes) but performance goes down as the size of the network increases. In contrast, the greedy algorithms (GDS, GIES) perform well for smaller networks but suffer from lower precision on larger networks. “Learn and Vote” outperformed the other methods in five out of nine studies in terms of F1 score, and is more stable even when the size of the network increases. For very small networks (i.e., fewer than 10 nodes), the PC-based approach has good sensitivity, however, it leaves many of the arc directions ambiguous (Fig 5a).

### Sensitivity to threshold

**Fig 6:**
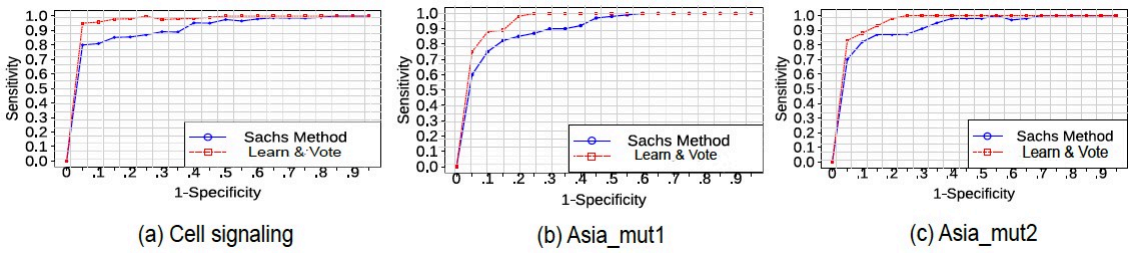
Sensitivity vs. FPR for “Learn and Vote” and the Sachs et al. method on three datasets: (a) Sachs et al. cell signaling; (b) Asia lung disease (mut1); and (c) Asia lung disease (mut2). The line plots are non-monotonic due to the use of different random initial DAGs for different points on the line plot.

In order to further analyze the sensitivity of our results with respect to the threshold parameter (in this study, set to 0.5) for predicting a causal arc, we compared the performance of “Learn and Vote” to that of the Sachs et al. method on three different network datasets (cell signaling, Asia_mut1, and Asia_mut2; see Methods and datasets) by plotting the sensitivity versus false positive error rate (FPR) for various threshold values (Fig 6a). On all three datasets, in terms of area under the sensitivity-vs-FPR curve, “Learn and Vote” has a higher score than the Sachs et al. method, with the most significant performance gap occurring at thresholds where the specificity is in the range of 0.7–0.9.

### Effect of sample size

**Fig 7:**
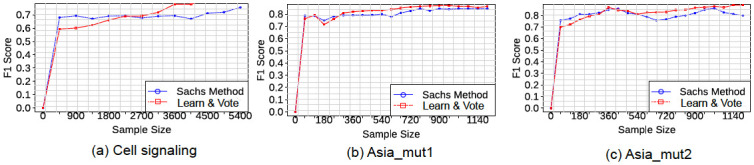
F1 vs. sample size for Learn and Vote and the Sachs et al. method, for three datasets.

It seems intuitive that in cases where single-experiment sample sizes are very small, separately analyzing data from individual experiments would be expected to perform poorly relative to a pooling-based approach like the Sachs et al. method. To test this, we analyzed the how the relative performances of “Learn and Vote” and the Sachs et al. method vary with sample size on the Sachs et al. dataset (for which the Sachs et al. method was specifically developed). We used sample of equal size from each experiment to prevent any bias towards a particular experiment. Fig 7 illustrates the performance of “Learn and Vote” in comparison to the Sachs et al. method by varying the sample size used from each experiment. When the number of samples per experiment is very small, using pooled data gives a better result. In case of the network Asia, which has eight nodes, when the number of samples per experiment is very small (e.g., 20 samples), the performance of “Learn and Vote” is not better than the pooling-based Sachs et al. method (Fig 7b and 7c). Hence, when only a small amount of data are available it is advantageous to pool them irrespective of how they may be derived from distinct experiments. However, if larger sample size is available, pooling appears to degrade the accuracy of network reconstruction.

## Discussion

To illustrate the pitfalls of the common practice of data pooling, we re-analysed the Sachs et al. dataset following their method of merging all the dataset from different experiments; while the analysis detected all the expected edges in the final causal network, it also generated many false positives (Fig 5f). Mindful that the ground-truth cell signaling network is likely incomplete (i.e., that there are likely latent interactions), we consulted the biological literature to investigate the false positive predicted arcs. The results of this post-analysis suggests that the interventions in the Sachs et al. study likely had “off-target” effects (Fig 5), consistent with the viewpoint that the perfect-interventions assumption is dubious for real-world molecular biology experiments. Pooling observations from multiple such datasets with different underlying distributions degrades precision of network reconstruction by–in essence–“noising” the causal structure. To improve precision for multi-experiment network inference when interventions are uncertain, we propose “Learn and Vote,” for which the key idea is to learn separately from each experiment and combine the resulting causal graphs. We tested our approach on five other synthetic networks after performing interventions on them to simulate experiments similar to the Sachs et al. study.

Taken together, our results (Fig 5 and Table 1) suggest that for analyzing datasets from studies that have imperfect interventions, greedy analysis methods (e.g., GDS, GIES, simy) are not as accurate as “Learn and Vote”. This could be because greedy methods pool all the data into one and try to pick up a locally optimal choice and hence do not perform well under uncertainty. On the other hand, due to its strict invariance property, the ICP method is conservative and reduces detection of false causal arcs to a great extent, but at the price of sensitivity (Fig 5d). The relatively poor performance of the PC method on the Sachs et al. dataset likely due the fact that it does not utilize knowledge about the targets of the interventions (i.e., it treats the data as observations only). In contrast, “Learn and Vote” uses a Bayesian approach with a robust optimizing network learning method which uses Tabu search along with the scoring metric (BDeu), which can be extended to add interventions, to make a grouped decision based on every experiments separately. Furthermore, to increase confidence in learning, we have also iterate the optimizing step 100 times with randomly initiated DAGs, for each experiment. The experiments were treated separately with respect to which nodes were intervened before we integrated the final graph. These steps resulted in a huge reduction of detection of false positives making “Learn and Vote” more accurate and robust than the other methods.

However, there are some cases where “Learn and Vote” poses challenges due to its current design. “Learn and Vote” works well if we have equal and adequate number of samples from each experiments. For cases where the number of samples per experiment is very small (as shown in Fig 7b and 7c), the performance of “Learn and Vote” is not better than the pooling-based method. Also, if samples collected from each experiment are unequal, the result with be biased towards experiments with more samples. These are difficult conditions to be fulfilled in a real world setting where experimentation is not in our control. Furthermore, “Learn and Vote” uses a score based method for causal learning, which are robust and works well with interventions, but are not scalable as network or data size increases, so the run-time of our method will increase with the network size. In future work, we plan to study the case of handling uneven samples of data from different experiments. We also plan to extend the work by choosing which interventional target is more informative in an unknown network structure. Another improvement of our approach is to see how choosing the number of random DAGs (we have taken 100) scales with network size. For example, in case of larger graphs, 100 might not be sufficient while in smaller graphs it could be overkill. One possible improvement to “Learn and Vote” would be an adaptive method for selecting the number of random initial DAGs; this is an area of planned future work. Also for cases where interventional data are available for a network inference application, we would investigate the effect of the ratio of observational to interventional data. For the synthetic network simulations in this study, we followed the Sachs et al. approach of having a significant ratio of interventional data to observation (7:2), although the ideal ratio may depend on the application domain. Ideally *n* − 1 experiments are sufficient (or in worst case necessary) to infer all the causal relations among *n* variables in a data [41]. So, we should intervene at all possible nodes. However, we have seen that intervening at leaf nodes are not useful, but that can be only done if the structure of the graph is already known. The correct proportions of intervention to observational experiment is a research question we want to explore in future, but we can conclude (from Fig 6, with Asia_mut2 having more interventions than Asia_mut1) that the more interventions we can perform, the better.

The current implementation of “Learn and Vote” takes averaging approach of weights learnt from each experiment. This, however, can make the method susceptible to become sensitive towards any extreme values or noise, if present. In that case another alternative of averaging could be majority voting where we can vote edges with more than 50% probabilities as 1, and use the majority votes as edges present in the final causal network. This could result in lesser edge in out output but will be more robust.

## Conclusion

We report a new approach, “Learn and Vote,” for learning a causal network structure from multiple datasets generated from different experiments, including the case of hybrid observational-interventional datasets. Our approach assumes that each dataset is generated by an unknown causal network altered under different experimental conditions (and thus, that the datasets have different distributions). Manipulated distributions imply manipulated graphs over the variables, and therefore, combining them to learn a network might increase statistical power but only if it assumes a single network that is true for every dataset. Unfortunately, this is not always the case under uncertain interventions. Our results are consistent with the theory that simply pooling measurements from multiple experiments with uncertain interventions leads to spurious changes in correlations among variables and increases the rate of false positive arcs in the consensus network. In contrast, our “Learn and Vote” method avoids the problems of pooling by combining experiment-specific weighted graphs. We compared “Learn and Vote” with six other causal learning methods on observational and interventional datasets having uncertain interventions. We found that for most of the larger-network datasets that we analyzed, “Learn and Vote” minimizes detection of false positive interactions and performs well in terms of F1 score. However, for cases where sample size per experiment is very small, we found that pooling works better. Our findings (i) motivate the need to focus on the uncertain and unknown effects of interventions in order to improve causal network learning precision, and (ii) suggest caution in using causal learning algorithms that assume perfect interventions, in the context of real world domains that have uncertain intervention effects.
